# Identifying High‐Performance Advanced Practice Profile of Specialist Nurses in Mainland China: A Mixed‐Methods Sequential Explanatory Study

**DOI:** 10.1155/jonm/3528145

**Published:** 2026-02-25

**Authors:** Wenjuan Zhao, Jie Zhong, Xiaobin Lai, Quan Cheng, Zheng Zhu, Yuxia Zhang

**Affiliations:** ^1^ Department of Nursing, Fudan University Shanghai Cancer Center, Shanghai, China, shca.org.cn; ^2^ Department of Oncology, Shanghai Medical College, Fudan University, Shanghai, China, fudan.edu.cn; ^3^ School of Nursing, Li Ka Shing Faculty of Medicine, The University of Hong Kong, Hong Kong, China, hku.hk; ^4^ School of Nursing, Fudan University, Shanghai, China, fudan.edu.cn; ^5^ Department of Neurosurgery, Xiangya Hospital, Central South University, Changsha, Hunan, China, csu.edu.cn; ^6^ National Clinical Research Center for Geriatric Disorders, Xiangya Hospital, Central South University, Changsha, Hunan, China, csu.edu.cn; ^7^ Department of Nursing, Zhongshan Hospital, Fudan University, Shanghai, China, fudan.edu.cn

**Keywords:** advanced practice nursing, latent profile analysis, mixed-methods design

## Abstract

**Objectives:**

Identifying high‐performing advanced practice nursing roles and understanding the factors that contribute to their effectiveness are critical for advancing professional development, optimizing workforce deployment, and ensuring long‐term sustainability in nursing. This study aimed to (1) identify distinct latent profiles of advanced practice nursing among specialist nurses in mainland China, (2) quantitatively examine the individual and contextual factors associated with high performance, as characterized by these profiles, and (3) qualitatively confirm the significant factors using explanatory semistructured interviews in the high‐performance groups.

**Methods:**

A mixed‐methods sequential explanatory design was used, in which quantitative data were collected first and subsequently explained through qualitative interviews. Certified specialist nurses from 16 hospitals across urban and rural areas of Shanghai were included. Latent profile analysis (LPA) was conducted using the five domains from the Advanced Practice Role Delineation tool as manifest indicators to classify nurses into distinct performance profiles. Multinomial logistic regression was used to examine potential determinants (e.g., job position) of group membership. Additionally, a backpropagation neural network (BPNN) was developed to rank the importance of contributing factors. Specialist nurses identified as high performers in the quantitative phase were purposively sampled for explanatory semistructured qualitative interviews.

**Results:**

Three latent profiles emerged: high performance (26.1%), moderate performance (46.3%), and low performance (27.6%). Compared to APNs, staff nurses had significantly lower odds of belonging to the high‐performance group (*β* = −1.715, *p* < 0.001). Nurses with higher professional career ladder (e.g., Level 4) were more likely to be in the high‐performance group (*β* = −1.163, *p* = 0.042). BPNN analysis identified the professional career ladder and job position as the most influential predictors of high performance. Qualitative findings from interviews with 17 participants reinforced these results, highlighting contextual factors such as leadership support (e.g., formal APN designation, physician endorsement, and organizational recognition) and individual attributes including specialized knowledge, extensive clinical experience, and advanced training.

**Conclusion:**

Identifying the profiles of advanced practice nursing roles provides valuable insights for optimizing APN performance and informing targeted management and policy strategies. High‐performing specialist nurses are positioned at the nexus of individual capability, interdisciplinary collaboration, and institutional support.

## 1. Introduction

Healthcare systems worldwide face growing pressure to deliver high‐quality care while simultaneously optimizing the expertise of healthcare providers and containing escalating costs [1, 2]. In response, many countries have expanded advanced practice roles for nurses. A growing body of evidence demonstrates the positive impact of advanced practice nursing on patient outcomes, including improved survival rates and quality of life, reduced hospitalizations and readmissions, lower healthcare expenditures, and enhanced equity in healthcare access [3].

There is consensus that advanced practice nursing goes “beyond basic practice within the clinical domain” distinguished by specialization, role advancement, and scope expansion [4]. Advanced nursing practice extends beyond direct bedside care to encompass domains that support and enhance the clinical environment, ultimately improving patient outcomes [5]. Research has shown that higher performance levels among advanced practice nursing roles are associated with greater professional autonomy [6, 7], which, in turn, contributes to enhanced job satisfaction and improved recruitment and retention. More importantly, higher performance levels have been linked to better patient outcomes such as reduced length of stay and decreased cost of care for hospitalized patients [8]. Therefore, identifying high‐performing advanced practice nursing roles and investigating the factors contributing to their effectiveness are essential to optimizing role development, utilization, and workforce sustainability.

To define, measure, and compare advanced practice nursing roles, various frameworks have been developed. Among these, the Strong Model of Advanced Practice is one of the most widely adopted [9]. According to this model, advanced practice includes five domains: direct care, support of systems, education, research, and professional leadership. First, as a large portion of advanced practice roles, the direct comprehensive care domain consists of patient‐focused activities including procedures, assessments, interpretation of data, and patient counseling [10]. Second, the support of systems domain incorporates activities that optimize functioning of the institution and includes recruitment and retention activities, strategic planning, mentoring, and quality improvement activities [11]. Third, the education domain encompasses a wide scope for education to meet the needs of patients, communities, clinicians, and students [12]. The fourth domain in the Strong Model, research, is aimed at supporting a culture of practice that challenges norms and seeks to improve patient outcomes through scientific enquiry [9, 12]. The last domain of professional leadership reflects a commitment to the profession, aiming at promoting nursing and healthcare, and includes disseminating nursing knowledge, serving as a resource/member in professional organizations, and acting as a consultant to individuals and groups [10].

Based on the Strong Model, the Advanced Practice Role Delineation (APRD) tool has been modified, validated, and utilized to delineate and define advanced practice nursing in Western [13–16] and Asian countries or regions [17–19]. Using this tool, previous quantitative studies distinguished advanced practice skills between advanced practice roles (e.g., specialist nurses, clinical nurse specialists, nurse practitioners, and advanced practice nurses [APNs]) and general staff nurses [13–16]. Additionally, previous studies have explored different levels of factors associated with advanced practice domains. Studies showed that at the institution level, the designated position of advanced practice nursing roles and professional career ladder emerged as particularly influential predictors of performance domains [20]. Role positions and specialty certification were modest positive predictors of involvement in all domains of advanced practice [15]. At the individual level, educational background was associated with performance across performance domains, particularly in providing direct care and education [5]. The other study supported significant differences appeared in education and age across all five performance domains [16]. This relationship was significant for the domains on research, support of systems, education, and leadership, but not for direct care [21]. In sum, the APRD tool has been adopted intensively to describe advanced practice performance and explore its associated factors across countries.

However, these prior quantitative studies have largely used variable‐centered approaches. While valuable for identifying average relationships between variables, such approaches inherently overlook the potential heterogeneity within the advanced practice nursing workforce. Specifically, they fail to capture the existence of distinct subgroups characterized by unique patterns of performance across multiple domains. Effective advanced practice nursing depends not solely on excelling in individual domains but on integrating clinical care, system‐level contributions, education, research, and leadership into a cohesive role to achieve optimal outcomes for patients, institutions, and the profession [22]. Latent profile analysis (LPA) offers a complementary, person‐centered approach that directly addresses this gap by identifying these meaningful subgroups (profiles) based on their performance patterns across all domains [23]. It is important to clarify that adopting LPA does not aim to replace the APRD tool, but rather to complement it. The key distinction lies in shifting from measuring performance across individual domains to identifying holistic performance profiles. This typological perspective not only facilitates the identification of high‐performing profiles but also supports the examination of underlying factors that contribute to such performance, thereby informing professional development and workforce strategies.

Mainland China has also explored the implementation of advanced practice nursing roles to meet the demands of a rapidly aging population and increasing healthcare complexity [24]. In Mainland China, the predominant advanced practice nursing role is that of the *specialist nurse*—a registered nurse who has completed systematic training in a specialized field, passed relevant certification exams, and possesses advanced clinical knowledge and skills to perform complex nursing interventions [25]. The China Nursing Development Outline (2005–2010) explicitly called for the cultivation of specialist nurses to raise clinical standards [26]. Although specialist nurses who have undergone systematic training can indeed meet current clinical needs and provide higher‐quality care, they lack a standardized regulatory framework. There is significant diversity in the educational backgrounds and job responsibilities among these nurses, particularly given that a large number do not meet the master’s degree requirement set by the International Council of Nurses (ICN) [27]. As a result, the role definitions for specialist nurses in China continue to face significant challenges and controversy. More urgently, research exploring the factors influencing performance of specialist nurses in China remains scarce, and evidence for enhancing their performance is similarly limited.

Additionally, according to Brown’s Framework for Advanced Practice Nursing (1998), APN roles are shaped by both individual competencies and contextual factors [28]. Yet, few studies have employed in‐depth qualitative methods to explore how these determinants interact at the individual and contextual level to influence performance [29–31]. However, qualitative evidence has been limited in the Chinese context. Therefore, the objectives of this study were to (1) identify distinct latent profiles of advanced practice nursing among specialist nurses in mainland China, (2) quantitatively examine the individual and contextual factors associated with high performance, as characterized by these profiles, and (3) qualitatively confirm the significant factors using explanatory semistructured interviews in the high‐performance groups.

## 2. Methods

### 2.1. Study Design

This study used a mixed‐methods sequential explanatory design [32], consisting of two phases: (1) a cross‐sectional survey of specialist nurses to identify latent high‐performance profiles and examine contributing factors and (2) explanatory semistructured qualitative interviews with selected nurses to explore their experiences and perceptions in depth. Integration occurred at multiple stages: The selection of interview participants was informed by the survey results, and the interview guide was developed based on quantitative findings to further explore key issues. The results from both phases were integrated during interpretation to generate a comprehensive understanding of high performance of advanced practice nursing role. Ethical approval was obtained from the Ethics Committee of Zhongshan Hospital, Fudan University (IRB: B2021‐004ER).

### 2.2. Settings and Participants

The study was conducted in 16 hospitals located in both urban and rural areas of Shanghai. Participants were certified specialist nurses who had completed formal training programs recognized by the government and were currently employed as staff nurses, clinical nurse managers, or officially designated APNs. All clinical nurse managers included in this study maintained direct patient care responsibilities as part of their advanced practice role. We adopted a census‐based approach rather than sampling, aiming to include the entire population of eligible specialist nurses in the study settings. The inclusion criteria were (1) completion of certified specialist nurse training and (2) voluntary informed consent to participate. The exclusion criteria included (1) working in nonclinical departments, (2) temporary training or exchange status, and (3) being on leave or away for external training during the survey. For the qualitative phase, we used purposive sampling to select participants from those identified as belonging to the high‐performance profile in the quantitative phase. Only specialist nurses who were classified in this subgroup and consented to be interviewed were considered. Within this group, we selected individuals with diverse characteristics to ensure maximum variation and enrich data interpretation.

### 2.3. Data Collection

From April to June 2019, we conducted an online cross‐sectional survey. After obtaining institutional approvals, local coordinators at each hospital distributed the survey via QR code following standardized training. Participation was voluntary, and only respondents who provided electronic informed consent could proceed. Data were collected and managed in real time using the Questionnaire Star system.

From July to October 2019, qualitative participants were selected, and data saturation guided the final sample size. All interviews were conducted one‐on‐one in private settings to ensure confidentiality and minimize distractions. Informed consent and permission for audio recording were obtained before each session.

### 2.4. Measurements

#### 2.4.1. Demographics and Institutional Variables

A self‐developed questionnaire captured institutional variables (e.g., hospital grade, type, and working department) and personal characteristics (e.g., age, gender, clinical position, professional career ladder, highest education background, and work experience). There are five levels of national professional career ladder in China, including Level 1 (junior nurse with qualification certificate), Level 2 (senior nurse), Level 3 (department‐level specialist/supervisor), Level 4 (senior expert/leadership role), and Level 5 (highest clinical expert). In this study, we used this professional career ladder to indicate the level of clinical profession in China.

#### 2.4.2. Chinese Version of APRD Tool

Based on the Strong Model of Advanced Practice [10], the APRD tool is a self‐reported questionnaire that includes five domains: clinical care, optimizing health systems, education, research, and leadership [33]. Each domain encompasses specific advanced practice activities. This tool has been widely used across countries including Finland, New Zealand, Spain, Saudi Arabia, and Singapore. The APRD tool was translated and validated for use in Chinese [34]. The Chinese version includes 37 items: clinical care (*n* = 13), optimizing healthcare systems (*n* = 8), education (*n* = 5), research (*n* = 5), and leadership (*n* = 6). Responses are rated on a 5‐point Likert scale (0 = Not at All to 4 = to a Very Great Extent). Domain scores were averaged, with higher scores indicating stronger role performance. It demonstrated strong psychometric properties: Cronbach’s alpha = 0.889; test–retest reliability = 0.982; I‐CVI = 0.80–1.00; and confirmatory factor analysis showed good fit [34].

#### 2.4.3. Qualitative Interview Guide

The interviews were led by a doctoral researcher (Wenjuan Zhao). Two pilot interviews were conducted to refine the guide. The core question was “What factors contribute to your outstanding performance?” Follow‐up questions included (1) What do you perceive as the main facilitators of your high performance? (2) What are the contextual factors (e.g., the designation of an advanced practice role) that affect your performance? (3) What personal or professional traits contribute to your effectiveness?

### 2.5. Data Analysis

#### 2.5.1. Quantitative Data Analysis

Descriptive statistics were used to summarize participant characteristics. Means and standard deviations were reported for continuous variables and frequencies and percentages for categorical variables. LPA was conducted using the five domains of the APRD tool as manifest indicators to classify specialist nurses into distinct performance profiles. Model fit was assessed using multiple complementary criteria. Information‐based indices included the *Akaike Information Criterion (AIC)*, *Bayesian Information Criterion (BIC)*, and *sample-size adjusted BIC (aBIC)*, where lower values indicate a better fit. For comparing nested models, the *Lo–Mendell–Rubin adjusted likelihood ratio test (LMR-LRT)* and *bootstrapped likelihood ratio test (BLRT)* were used, with statistically significant results supporting the more complex model. *Entropy* values greater than 0.80 were considered indicative of good classification quality. Final model selection was based on a combination of statistical indicators, theoretical interpretability, and practical considerations, such as ensuring that each profile comprised at least 5% of the sample to ensure meaningful representation. Differences in demographic and institutional characteristics across latent profiles were examined using *chi-square* tests. *Fisher’s* exact test was applied for variables with expected cell counts less than 5. To identify predictors of profile membership, multinomial logistic regression was performed, with the dependent variable being profile category and independent variables selected based on significance in univariate analyses. To further assess the relative importance of associated factors, a *backpropagation neural network (BPNN)* model was developed.

#### 2.5.2. Qualitative Data Analysis

All interview recordings were transcribed verbatim within 24 h. Two researchers (Wenjuan Zhao and Xiaobin Lai) independently verified transcript accuracy, and transcripts were returned to participants for member checking to enhance data trustworthiness. Transcripts were organized and managed using MAXQDA 2020 (VERBI Software, Germany). Each participating nurse was named N1 to N17 in the coding system without identifications. Thematic analysis was conducted to identify patterns across participants’ narratives while preserving contextual meaning. This analysis followed a structured yet flexible process, beginning with transcript organization and systematic coding, followed by analytic memo development, identification and refinement of thematic patterns, synthesis of findings, and integration with the existing literature [35]. Coding was conducted independently by two researchers in Chinese, and discrepancies were resolved through discussion with the research team. Codes, themes, and illustrative quotations were then translated into English by Jie Zhong and verified by Wenjuan Zhao and Xiaobin Lai.

#### 2.5.3. Integration

Data integration was incorporated into the study design, data collection, and interpretation phases. A sequential explanatory approach guided the process, with the qualitative phase designed to elaborate on contextual and individual‐level factors identified in the quantitative phase. During data collection, interview participants were purposively selected from the high‐performance subgroup identified through LPA. Interview questions were informed by quantitative results to further investigate relevant factors including the contextual factors (e.g., designation of APN position) and individual factors (e.g., education background and professional career ladder). In the interpretation phase, a narrative approach was adopted to organize and present qualitative insights in relation to the key contextual and individual determinants emerging from the quantitative analysis, thereby deepening the understanding of high performance in advanced nursing practice.

#### 2.5.4. Patient and Public Involvement

Patient and the public were not involved in the design, conduct, reporting, or dissemination plans of our research in nurses.

## 3. Results

### 3.1. Quantitative Results

#### 3.1.1. Participant Characteristics

A total of 889 questionnaires were collected. After excluding 138 responses due to ineligibility (*n* = 35), uniform response patterns (*n* = 84), or patterned answering behavior (*n* = 19), 751 valid responses were retained for analysis, yielding an effective response rate of 84.5%. Participant characteristics are presented in Table 1. The sample predominantly consisted of female nurses (97.3%), with most employed in tertiary (79.4%) and general hospitals (84.6%), reflecting the distribution of specialist nurses in urban China. The mean age was 35.07 years (SD = 6.01), with an average of 14.54 years (SD = 6.99) of clinical experience and 8.71 years (SD = 6.25) in the current position. Most respondents held a bachelor’s degree (71.6%), and most were classified as Level 2 nurses (53.7%) or Level 3 nurses (37.2%). Nearly half worked in critical or emergency care departments (47.9%), followed by medical/surgical units (35.6%). Regarding employment position, 65.4% were staff nurses, 16.5% were clinical nurse managers, and 18.1% were APNs.

**Table 1:** Demographic characteristics of participants by practice profile (*N* = 751)

Variables	Categories	Overall (*N* = 751)	Low performance (*N* = 207)	Moderate performance (*N* = 348)	High performance (*N* = 196)	χ^2^	*p* values
		*N* (%)	*N* (%)	*N* (%)	*N* (%)		
Hospital grade	Secondary	155 (20.6)	45 (21.7)	78 (22.4)	32 (16.3)	3.048	0.218
	Tertiary	596 (79.4)	162 (78.3)	270 (77.6)	164 (83.7)		

Hospital type	General	635 (84.6)	183 (88.4)	294 (84.5)	158 (80.6)	4.685	0.096
	Specialty	116 (15.4)	24 (11.6)	54 (15.5)	38 (19.4)		

Working department	Women and children	97 (12.9)	17 (8.2)	54 (15.5)	26 (13.3)	17.014	0.030
	Critical care	360 (47.9)	119 (57.5)	149 (42.8)	92 (46.9)		
	Internal medicine and surgery	267 (35.6)	67 (32.4)	132 (37.9)	68 (34.7)		
	Oral and ENT	12 (1.6)	2 (1.0)	7 (2.0)	3 (1.5)		
	Other	15 (2.0)	2 (1.0)	6 (1.7)	7 (3.6)		

Gender	Male	20 (2.7)	4 (1.9)	5 (1.4)	11 (5.6)	9.022	0.011
	Female	731 (97.3)	203 (98.1)	343 (98.6)	185 (94.4)		

Age	≤ 25 years	19 (2.5)	7 (3.4)	7 (2.0)	5 (2.6)	12.557	0.051
	25–35 years	417 (55.5)	129 (62.3)	175 (50.3)	113 (57.7)		
	35–45 years	248 (33.0)	55 (26.6)	127 (36.5)	66 (33.7)		
	> 45 years	67 (8.9)	16 (7.7)	39 (11.2)	12 (6.1)		

Years of experience as a nurse	≤ 3 years	22 (2.9)	6 (2.9)	10 (2.9)	6 (3.1)	7.774	0.456
	3–5 years	29 (3.9)	9 (4.3)	14 (4.0)	6 (3.1)		
	5–10 years	206 (27.4)	68 (32.9)	80 (23.0)	58 (29.6)		
	10–20 years	340 (45.3)	85 (41.1)	167 (48.0)	88 (44.9)		
	> 20 years	154 (20.5)	39 (18.8)	77 (22.1)	38 (19.4)		

Years of experience in the current position	≤ 3 years	170 (22.6)	47 (22.7)	83 (23.9)	40 (20.4)	7.689	0.464
	3–5 years	117 (15.6)	39 (18.8)	50 (14.4)	28 (14.3)		
	5–10 years	221 (29.4)	62 (30.0)	95 (27.3)	64 (32.7)		
	10–20 years	198 (26.4)	48 (23.2)	94 (27.0)	56 (28.6)		
	> 20 years	45 (6.0)	11 (5.3)	26 (7.5)	8 (4.1)		

Professional career ladder	Level 1	45 (6.0)	17 (8.2)	17 (4.9)	11 (5.6)	51.589	< 0.001
	Level 2	403 (53.7)	144 (69.6)	181 (52.0)	78 (39.8)		
	Level 3	279 (37.2)	44 (21.3)	141 (40.5)	94 (48.0)		
	Level 4	24 (3.2)	2 (1.0)	9 (2.6)	13 (6.6)		

Highest education	Associate degree and below	195 (26.0)	66 (31.9)	90 (25.9)	39 (19.9)	37.054	< 0.001
	Bachelor’s degree	538 (71.6)	141 (68.1)	255 (73.3)	142 (72.4)		
	Master’s degree	18 (2.4)	0 (0.0)	3 (0.9)	15 (7.7)		

Position	Staff nurse	491 (65.4)	183 (88.4)	217 (62.4)	91 (46.4)	84.733	< 0.001
	Clinical nurse manager	124 (16.5)	9 (4.3)	70 (20.1)	45 (23.0)		
	Advanced practice nurse	136 (18.1)	15 (7.2)	61 (17.5)	60 (30.6)		

#### 3.1.2. Latent Profile Identification

LPA models with one to four latent classes were tested and compared. Fit indices for each model are shown in Table 2. The three‐class model was selected as the optimal solution based on several criteria. First, it demonstrated lower AIC, BIC, and aBIC values compared to one‐ and two‐class models. Second, it showed acceptable entropy values (> 0.80), indicating high classification accuracy. Third, although the four‐class model showed marginally better AIC/BIC values, the LMR‐LRT (*p* > 0.05) suggested no significant improvement over the three‐class model. Discriminant analysis further supported the classification accuracy, with correct classification rates of 93.8%, 92.1%, and 92.6% for Classes 1, 2, and 3, respectively (Table 3). Comparative analyses of the five APRD domains across the three latent profiles revealed statistically significant differences (*p* < 0.05) for all domains (Table 4).

**TABLE 2:** Fit indices for competing latent profile models (*N* = 751).

Models	AIC	BIC	aBIC	Entropy	*p* values		Class percentages (%)
					LMR	BLRT	
One‐profile	9680.306	9726.52	9694.766	—	—	—	—
Two‐profile	8365.265	8439.207	8388.401	0.821	< 0.001	< 0.001	0.48/0.52
Three‐profile	7860.721	7962.392	7892.533	0.843	< 0.001	< 0.001	0.28/0.46/0.26
Four‐profile	7734.084	7863.483	7774.572	0.822	0.0857	< 0.001	0.20/0.33/0.10/0.37

*Note:* aBIC = adjusted BIC; LMR‐LRT = Lo–Mendell–Rubin adjusted likelihood ratio test.

Abbreviations: AIC, Akaike Information Criterion; BIC, Bayesian Information Criterion; BLRT, bootstrapped likelihood ratio test.

**TABLE 3:** Average posterior probabilities for latent profile membership.

Latent classes	Discriminate analysis		
	Class 1	Class 2	Class 3
Class 1	0.938	0.062	< 0.001
Class 2	0.036	0.921	0.043
Class 3	< 0.001	0.074	0.926

*Note:* Class 1, low‐performance group; Class 2, moderate‐performance group; Class 3, high‐performance group. Values represent the average posterior probabilities of correctly classifying individuals into each latent profile. Higher diagonal values (≥ 0.90) indicate strong classification precision.

**TABLE 4:** Distribution of advanced practice nursing performance in the three‐profile model (*N* = 751), mean (SD).

Dimensions	Total sample (*n* = 751)	Low performance (*n* = 207)	Moderate performance (*n* = 348)	High performance (*n* = 196)	*F*	*p* values
Clinical care	2.73 ± 0.65	2.31 ± 0.61	2.68 ± 0.53	3.26 ± 0.50	153.016	< 0.001
Optimizing health systems	2.14 ± 0.92	1.07 ± 0.55	2.20 ± 0.48	3.16 ± 0.48	889.683	< 0.001
Education	2.49 ± 0.88	1.50 ± 0.64	2.55 ± 0.49	3.42 ± 0.46	674.268	< 0.001
Research	1.85 ± 0.98	0.74 ± 0.50	1.88 ± 0.53	2.99 ± 0.53	939.917	< 0.001
Leadership	1.01 ± 1.01	0.17 ± 0.37	0.96 ± 0.78	1.98 ± 1.00	283.612	< 0.001

**FIGURE 1:**
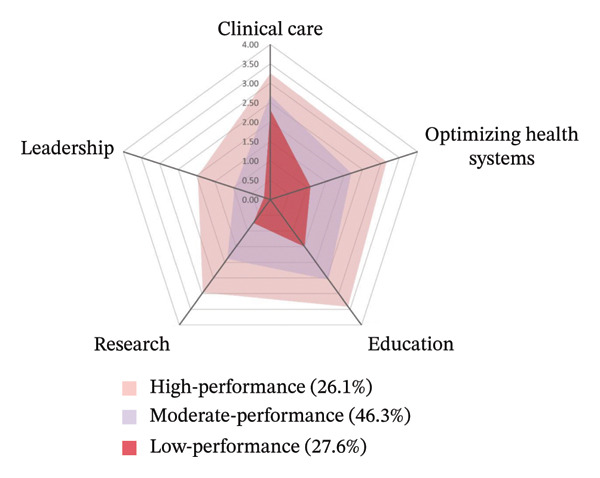
Latent profiles of advanced practice nursing performance.

Based on scores across the five APRD domains, the latent profiles were labeled as follows: Class 1 (27.6%) had the lowest scores across all domains and was labeled the low‐performance profile, with clinical care (2.31 ± 0.61) as the highest‐scoring domain, followed by education (1.50 ± 0.64), optimizing health systems (1.07 ± 0.55), research (0.74 ± 0.50), and leadership (0.17 ± 0.37). Class 2 (46.3%) showed moderate scores and retained the same domain ranking pattern as Class 1, hence labeled the moderate‐performance profile. Class 3 (26.1%) demonstrated the highest scores across all domains and was labeled the high‐performance profile, with education scoring highest (3.42 ± 0.46), followed by clinical care (3.26 ± 0.50), optimizing health systems (3.16 ± 0.48), research (2.99 ± 0.53), and leadership (1.98 ± 1.00) (Figure 1).

#### 3.1.3. Associated Factors of Latent Profiles

**FIGURE 2:**
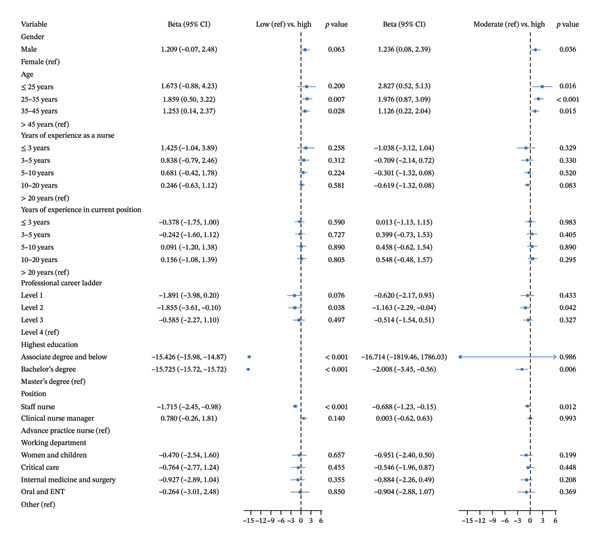
Multinomial logistic regression of factors associated with latent profile membership.

Statistically significant differences were observed across the three profiles in terms of working department, sex, professional career ladder, highest education, and position (Table 1). A multivariate logistic regression model was constructed using the variables with statistically significant differences as determined by *χ* ^2^ tests and age, working experience decided by professional judgment. The results are shown in Figure 2.

##### 3.1.3.1. Low‐Performance vs. High‐Performance Profiles

Middle‐aged nurses (25–35 years: *β* = 1.859, *p* = 0.007; 35–45 years: *β* = 1.253, *p* = 0.028) were more likely than older nurses (> 45 years) to be in the high‐performance profile. Nurses with lower professional career ladder (e.g., Level 2) were less likely to belong to the high‐performance profile compared to those with higher ladder (e.g., Level 4; *β* = −1.855, *p* = 0.038). Lower educational attainment was also negatively associated with high performance (associate degree and below vs. master’s and above: *β* = −15.426, *p* < 0.001; bachelor’s vs. master’s and above: *β* = −15.725, *p* < 0.001). Staff nurses were significantly less likely to be in the high‐performance profile than APNs (*β* = −1.715, *p* < 0.001).

##### 3.1.3.2. Moderate‐Performance vs. High‐Performance Profiles

Male nurses were more likely than females to be in the high‐performance profile (*β* = 1.236, *p* = 0.036). Younger nurses also had higher odds of being in the high‐performance profile (≤ 25 years: *β* = 2.827, *p* = 0.016; 25–35 years: *β* = 1.976, *p* < 0.001; 35–45 years: *β* = 1.126, *p* = 0.015). Lower career ladder (Level 2 vs. Level 4: *β* = −1.163, *p* = 0.042) and lower educational levels (bachelor’s vs. master’s and above: *β* = −2.008, *p* = 0.006) were again associated with reduced likelihood of high performance. Compared to APNs, staff nurses were less likely to belong to the high‐performance profile (*β* = −0.688, *p* = 0.012).

#### 3.1.4. Ranking of Contributing Factors

**FIGURE 3:**
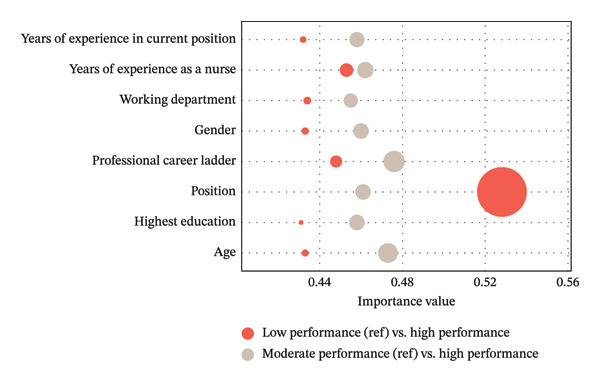
Importance values of factors associated with practice profile classification in the backpropagation neural network.

The BPNN model was used to rank the importance of the eight key factors identified in the logistic regression models. For the low‐performance vs. high‐performance comparison, the most influential factors in the descending order were position, years of nursing experience, professional career ladder, department, sex, age, years in the current position, and education level (the importance values are 0.528, 0.453, 0.448, 0.434, 0.433, 0.433, 0.432, and 0.431). For the moderate‐performance vs. high‐performance comparison, the order of importance was professional career ladder, age, years of nursing experience, position, sex, years in the current position, education level, and department (the importance values are 0.476,0.473, 0.462, 0.461, 0.460, 0.458, 0.458, and 0.455) (Figure 3).

### 3.2. Qualitative Results

#### 3.2.1. Participant Characteristics

A total of 17 specialist nurses participated in the qualitative phase. All interviews were completed in full, with durations ranging from 31 to 61 min (mean: 42.18 ± 8.09 min). Among the participants, two were male and 15 were female. The participants’ ages ranged from 30 to 48 years (mean: 35.65 ± 5.72 years), with total clinical experience ranging from 8 to 30 years (mean: 14.24 ± 7.06 years), and experience in their current positions ranging from 3 to 16 years (mean: 6.88 ± 4.05 years). The nurses’ professional career ladder levels comprised 3 at Level 4, 9 at Level 3, and 5 at Level 2. Regarding educational background, two participants held doctoral degrees, two held master’s degrees, and 13 held bachelor’s degrees.

#### 3.2.2. Contextual Leadership Support

Quantitative findings identified job position as the most influential predictor of high performance. Consistent with this, qualitative interviews emphasized the role of contextual leadership support in facilitating advanced practice nursing.

##### 3.2.2.1. Designation of APN Position

Participants noted that the formal designation of advanced practice nursing roles accelerated professional development by enabling nurses to accumulate high volumes of relevant clinical experience and refine advanced competencies in a structured manner. N1, a vascular access specialist, explained: “*This allows them to encounter numerous relevant cases in a short period, quickly mastering diagnosis and management of vascular access-related issues. This is a major advantage of specialization—concentrated exposure to high patient volumes accelerates competency development*.” N9 added, “*Because of our focused role, we could immediately mobilize resources to ensure a better care plan for their next visit—potentially altering their entire treatment trajectory. This is critical. Full-time specialization inevitably yields better results than part-time efforts*.” The importance of full‐time role commitment was also highlighted. N4 shared, “*As a full-time APN, I constantly review guidelines, seek learning opportunities, and encounter complex cases*.” N9 echoed this, noting, “*Only when you dedicate your full time to this role can you develop depth. Accumulated experience leads to breakthroughs*.”

##### 3.2.2.2. Physician Acceptance: Establishment of Professional Authority

Physician endorsement was seen as essential for establishing professional authority. Although APNs lacked prescriptive authority, many reported that physicians respected their clinical judgment. N7 emphasized the dual role of physician and leadership support in facilitating implementation: “*When we consult physicians on clinical issues, they provide valuable medical input and guidance. This support is critical - if they see value in our role, implementation becomes much smoother. Additionally, leadership support is essential because frontline nurses may resist changes that disrupt their routines or increase workload. Nurse managers or department heads must reinforce the importance of APN roles; otherwise, clinical adoption becomes difficult*.” This trust extended beyond formal authority, as N1 described: “*Although we still lack prescriptive authority and cannot directly order tests, physicians trust our clinical judgment. After discussing with us, they often act on our recommendations for managing catheter-related complications*.”

##### 3.2.2.3. Institutional Recognition: Sustainability of APN Practice

Formal institutional recognition was critical for legitimizing APN roles and integrating them into hospital systems. Participants noted that official appointments enhanced their credibility, facilitated interdisciplinary collaboration, and enabled them to perform operational tasks such as patient referrals. N17 explained: “*The hospital issued official APN appointment certificates, which enhanced our credibility. Physicians now actively seek collaboration with us*.” N3 added, “*Only after hospital approval could we formally refer patients and coordinate care—many workflows require institutional authorization. Without recognition, securing support is impossible*.” Institutions that tied APN accountability to performance metrics further reinforced autonomy and motivation. N14 stated, “*APNs never need nurse managers to assign tasks—they proactively identify their responsibilities. Their annual salary increases are tied to performance metrics, including task completion and overall contribution. Compensation is a key motivator for sustained engagement*.”

#### 3.2.3. Individual Professional Development

Quantitative analysis showed that higher professional career ladder and education level were associated with high‐performance profiles. The qualitative findings further highlighted three interrelated dimensions contributing to individual development: comprehensive specialty knowledge, substantial clinical experience, and advanced training and education.

##### 3.2.3.1. Comprehensive Specialty Knowledge

Participants emphasized the importance of maintaining an updated and in‐depth knowledge base. N10 noted, “*Specialty knowledge is crucial and must be continuously updated……When you can discuss professional knowledge fluently with physicians, they recognize you as trustworthy.” N2 commented, “APNs frequently assist physicians in treatment decision-making, patient education, and even procedures like lympho-vascular therapy. Their required knowledge scope and skill level significantly exceed that of general nurses - this demands deliberate knowledge preparation.*” Several participants also discussed their roles in knowledge creation and dissemination. N5 stated, “*Beyond clinical work, APNs need nursing science competencies to build systems. They must: master fundamental specialty knowledge, generate knowledge through research, and disseminate knowledge through education. These are equally vital for hospitals and universities.*”

##### 3.2.3.2. Substantial Clinical Experience

Extensive clinical experience was described as essential for advanced practice. Participants reported that their ability to recognize subtle changes in patient conditions, educate others, and lead interventions stemmed from repeated exposure to complex cases. N12 explained, “*APNs must have substantial clinical experience—often more vital than theoretical knowledge……guiding junior nurses, educating patients, or performing procedures stem from repeated practice.*” N11 observed, “*Inexperienced nurses struggle to command patient trust. It’s not those patients are difficult, but they instinctively question unfamiliar providers. If you’re not exceptionally skilled in your specialty, patients will doubt your competence.*”

##### 3.2.3.3. Advanced Training and Education

Quantitative results showed that advanced education (e.g., master’s and above) was strongly linked to high‐performance profiles. Interviewees confirmed that structured APN training equipped them with research, critical thinking, and evidence‐based practice skills. N11 shared, “*The structured APN training program has been tremendously helpful for me. I previously lacked systematic research knowledge, but through this training, I’ve developed a completely different skillset—I now possess methodological thinking and research competencies.*” Second, higher education equips APNs with critical academic skills that enable them to independently evaluate and apply the latest evidence, ensuring their practice remains aligned with current medical advancements. As N2 explained, “*access the latest specialty literature, stay updated on treatments, conduct literature reviews, and draft research proposals,*” with one participant noting that “*original ideas often correlate with educational background.*” Finally, the ability to engage in clinical research and knowledge generation was also viewed as a key advantage of graduate‐level education. N2 stated, “*Higher education helps because accessing the latest specialty literature, staying updated on treatments, conducting literature reviews, and even drafting research proposals require academic skills. Original ideas often correlate with educational background.*”

## 4. Discussion

### 4.1. Principal Findings on Latent Profiles

This study identified three distinct practice profiles among specialist nurses in Mainland China using LPA of self‐reported advanced practice activities across five domains. Based on the five advanced practice domains, our findings identified low‐performance profile (27.6%), moderate‐performance profile (46.3%), and high‐performance profile (26.1%). The largest group, labeled the moderate‐performance profile, exhibited moderate levels of performance, with strengths in clinical care and education. Although this pattern was similar to that of the low‐performance group (27.6%), the moderate‐performance group demonstrated higher proficiency, who likely represent the core workforce of specialist nurses in Chinese hospitals: clinically competent but lacking fully developed capabilities in system‐level domains such as optimizing health systems, research, and leadership. The low‐performance group, despite sharing a similar domain distribution, likely includes early‐career nurses or those working in settings with limited opportunities for broader professional development.

The most clinically significant finding emerged in the high‐performing group (26.1%). These specialists excelled most prominently in clinical care and education, forming an “expert educator–clinician” profile. Notably, this group demonstrated a unique pattern in which education surpassed clinical care as their primary activity, complemented by strong engagement in optimizing health systems, research, and leadership. This high‐performance profile exemplifies how Chinese APNs effectively integrate clinical practice with education, system improvement, research, and leadership, thereby delivering holistic, high‐quality care while driving innovation and reform within healthcare institutions [36]. Alongside clinical care, educational and mentoring activities constituted core responsibilities for Chinese specialist nurses, aligning with patterns observed in Hong Kong [18] and Australia [13]. This showed that APNs functioned both as clinical educators and as key facilitators of patient and family education. Despite their advanced competencies, leadership scores lagged significantly behind other dimensions—a finding that contrasts with Saudi Arabian APNs [19], who demonstrate more balanced role mastery. This discrepancy may stem from limitations in educational attainment, resource accessibility, and structured development opportunities. Most Chinese APNs lack postgraduate training [18], which limits exposure to leadership development frameworks. In addition, institutional constraints (e.g., time and administrative support) may hinder leadership development [37]. Finally, limited awareness of or access to avenues for influencing health policy and clinical practice may further restrict leadership expression [38].

### 4.2. Factors Associated With Higher Performance

Regarding factors associated with higher performance, quantitative findings identified job position, professional career ladder, and education background as the most influential predictors of higher performance. Consistent with these, explanatory qualitative interviews emphasized the role of contextual leadership support (e.g., designation of APN position) and individual professional development (e.g., comprehensive specialty knowledge) in facilitating advanced practice nursing.

Both quantitative and qualitative findings supported that the formal designation of an APN position was the most critical factor for progression from low‐ to high‐performing profiles. Compared to specialist nurses holding APN positions, those in staff nurse roles were significantly less likely to achieve high levels of advanced practice performance. This observation is consistent with international evidence. In Canada, Doran et al. (2014) demonstrated that nurses in formalized specialist roles exhibited greater autonomy and system‐level impact compared to general staff nurses [39]. Similarly, Sevilla Guerra et al. (2018) found that Spanish APNs with clearly defined positions were more likely to engage in role‐optimizing activities such as research and mentorship [20]. These cross‐national parallels reinforce the notion that obtaining a formally recognized position aligned with advanced practice competencies is a fundamental prerequisite for role development [15]. This finding highlights the pivotal role of formalized APN positions within China’s nursing career framework. Such positional authority enables APNs to identify system‐level challenges, implement specialized interventions, conduct clinical research, and mentor junior staff as activities that collectively enhance the standards of nursing practice and the quality of patient care. Additionally, our qualitative findings revealed that formal APN positions served as the cornerstone of advanced practicing performance through two fundamental mechanisms: structured competency development and interprofessional/institutional recognition. Physician acceptance plays a critical role in establishing the professional authority of APNs among patients and within healthcare institutions. A qualitative study involving primary care physicians indicated that they view APNs as competent healthcare professionals [40]. Existing evidence further suggests that strong working relationships, effective collaboration with physicians, and mutual trust within teams contribute significantly to both the autonomy and informal empowerment of APNs [37]. Conversely, overly authoritarian or bureaucratic interactions by physicians may undermine APN autonomy and impede, rather than support, independent practice [41]. These findings underscore the importance of system‐level recognition of APNs, particularly from physicians and interdisciplinary team members, in enhancing APN autonomy.

Apart from holding specialized positions, progression toward higher performance was largely influenced by individual development, specifically, education level, work experience or age (younger versus more experienced nurses), and professional career ladder (higher qualifications). Nurses with lower educational attainment faced significantly greater challenges in attaining high‐performance profiles, highlighting the importance of higher education in cultivating advanced practice competencies. Although a master’s degree is considered the standard qualification for APNs according to the ICN, variations exist across healthcare systems due to differing educational resources and infrastructures. In China, the educational prerequisites for entry into specialist nurse training programs vary considerably. Nearly one‐fifth (19.1%) of programs have no formal educational requirement, while most (63.9%) require at least a college diploma. A bachelor’s degree or higher is mandatory for 17.0% of programs [42]. Given this variability, the professional career ladder may serve as a more meaningful indicator of high‐performance advanced practice than education level alone in China. Our qualitative findings revealed that the professional career ladder encapsulates multiple dimensions of individual development, including extensive clinical experience, comprehensive specialty knowledge, and advanced training and education. While higher academic degrees were associated with improved performance, clinical experience and domain‐specific expertise were equally emphasized. Therefore, for Chinese specialist nurses aspiring to high performance, individual development is potentially within their own control and achievable through cumulative clinical growth and continuous learning.

However, unlike studies from Spain [20] and Australia [43], which reported no significant association between age or experience and the level of advanced practice nursing activity, our findings revealed that younger nurses with shorter work experience were more likely to attain high‐performance status compared to those aged over 45 years with more than 20 years of experience. Based on these findings, we speculate that early‐ or mid‐career nurses may exhibit greater adaptability to evidence‐based practice and evolving clinical models. In the context of China’s healthcare system, institutional support and developmental opportunities may also be preferentially directed toward younger nurses, whereas those with longer work histories are more likely to transition into nonclinical or administrative roles.

### 4.3. Implications for Clinical Practice and Health Policy

This mixed‐methods study has important implications for optimizing advanced practice nursing roles in resource‐limited healthcare systems where such roles are still in the early stages of development. First, our study in Chinese specialist nurses reveals that only the high‐performance profile aligns with international standards for advanced practice [1]. In contrast, the other two identified profiles do not meet these criteria. This suggests that in the absence of formal regulation, a high‐performance profile identified by tools such as the LPA may serve as a functional proxy for advanced practice recognition, while other profiles would not qualify under the same standard. Additionally, our findings indicate that high‐performing APNs function at the intersection of individual expertise (reflected by the higher professional career ladder), interdisciplinary collaboration (particularly physician acceptance), and institutional support or recognition. Educational attainment and work experience alone were not independently associated with high performance, whereas career ladder emerged as a significant predictor. Quantitative results identified the higher professional career ladder as the strongest factor associated with achieving a high‐performance profile. This suggests that only the combination of education and experience yields meaningful effects, and both components are indispensable. Accordingly, both academic qualifications and duration of clinical practice should be jointly required in the design of advanced practice nursing roles’ training and certification programs.

Furthermore, formal APN positions confer institutional recognition that strengthens APN autonomy in clinical practice. Autonomy is essential for APNs to provide care that aligns with their level of education and training. Both researchers and policymakers have increasingly advocated for the expansion of APN scope of practice and professional autonomy to support their enhanced role in healthcare delivery. In the USA, for example, 26 states have adopted full practice authority, allowing APNs to practice independently without physician supervision [44]. Under this model, APNs are licensed and authorized to evaluate patients, diagnose conditions, order and interpret diagnostic tests, and initiate and manage treatments, including the prescription of medications and controlled substances, under the authority of state nursing boards [45]. Collaboration between APNs and physicians remains a cornerstone of high‐quality, safe, and cost‐effective care [46], and APNs are held to the same standards as other clinicians in terms of billing and reimbursement procedures [47]. Although the advanced practice nursing system in China is still in its early stages, the presence of formal APN positions has already contributed to a greater sense of empowerment and confidence among APNs regarding their roles, responsibilities, and competencies in patient care. These findings strongly support policy initiatives aimed at expanding dedicated APN positions and establishing differentiated career pathways from general staff nurses—pathways that formally recognize and reward advanced practice competencies.

### 4.4. Limitations

This study has several limitations that should be noted. First, causal relationships could not be established due to the cross‐sectional design. Second, the overrepresentation of critical care specialists (47.9%) reflects the historical emphasis on critical care within China’s specialist nurse training programs, which may limit the applicability of findings to other specialties. Additionally, the underrepresentation of male nurses (2.7%) and nurses from secondary hospitals (20.6%) may restrict the generalizability of the results to the broader nursing population. Third, since the qualitative interviews were designed to explore the characteristics of high‐performing profiles, future qualitative or mixed‐methods studies could include participants from low‐performance profiles to examine important barriers faced by these subgroups.

## 5. Conclusion

This study identified subgroups of specialist nurses with distinct advanced practicing performance profiles and demonstrated that both contextual and individual characteristics varied significantly across these groups. Based on the results presented in this study, Chinese specialist nurses in the high‐performance profile appear to align with international standards for advanced practice. Findings from both the quantitative and qualitative phases consistently indicated that the formal designation of an APN position was the most critical factor in the progression from low‐ to high‐performing profiles. In addition, transitions to higher performance were influenced by individual professional development, including educational attainment, work experience or age, and career ladder. Overall, the results suggest that high‐performing APNs function at the intersection of individual expertise, physician acceptance, and institutional support. To further optimize the contributions of APNs, healthcare institutions must actively foster enabling environments that support research engagement and leadership development. This includes the formal recognition of APNs’ roles in evidence‐based practice, provision of protected time for scholarly activities, and implementation of structured interprofessional leadership opportunities. Key strategies should prioritize the establishment of credentialing systems, designation of formal APN positions, and the safeguarding of professional autonomy through clearly defined responsibilities and accountability structures. Future research is warranted to generate more rigorous evidence on the comprehensive, multilevel factors that contribute to high‐performing advanced practice among specialist nurses. Notably, the study sample was predominantly composed of staff nurses holding bachelor’s degrees, with the largest proportion aged between 25 and 35 years. This demographic profile highlights the pressing need to develop targeted retention strategies and supportive work environments that effectively harness and sustain the expertise of experienced specialist nurses.

## Author Contributions

Wenjuan Zhao and Jie Zhong contributed equally to the paper through conceptualization, data analysis, data interpretation, paper writing, and revision. Xiaobin Lai contributed to the paper through conceptualization, data interpretation, and paper revision. Quan Cheng contributed through data interpretation, result visualization, and paper revision. Zheng Zhu contributed through administration, data interpretation, and paper revision. Yuxia Zhang contributed through mentorship, conceptualization, and paper revision.

## Funding

This work was supported by the Nursing Research Sub‐Project of the China Health Talent Development Program (Grant No. 2018‐HLYJ‐013) and Fudan University Hospital Management Enhancement Project (Grant No. FDYGC20180201).

## Conflicts of Interest

The authors declare no conflicts of interest.

## Data Availability

The data that support the findings of this study are available on request from the corresponding author. The data are not publicly available due to privacy or ethical restrictions.

## References

[1] International Council of Nurses , ICN-International Council of Nurses, 2020.

[2] World Health Organization , State of the World’s Nursing 2020: Investing in Education, Jobs and Leadership, 2020.

[3] Rodríguez-García A. , Borrallo-Riego Á. , Magni E. , and Guerra-Martín M. D. , Effectiveness of Advanced Practice Nursing Interventions on Diabetic Patients: a Systematic Review, Healthcare. (2025) 13, no. 7. 10.3390/healthcare13070738 PMC11989214 40218036

[4] Dowling M. , Beauchesne M. , Farrelly F. , and Murphy K. , Advanced Practice Nursing: a Concept Analysis, International Journal of Nursing Practice. (2013) 19, no. 2, 131–140, 10.1111/ijn.12050, 2-s2.0-84876301701. 23577970

[5] Wilkinson J. , Carryer J. , and Budge C. , Impact of Postgraduate Education on Advanced Practice Nurse Activity – a National Survey, International Nursing Review. (2018) 65, no. 3, 417–424, 10.1111/inr.12437, 2-s2.0-85044245697. 29569420

[6] Miedaner F. , Kuntz L. , Enke C. , Roth B. , and Nitzsche A. , Exploring the Differential Impact of Individual and Organizational Factors on Organizational Commitment of Physicians and Nurses, BMC Health Services Research. (2018) 18, no. 1, 10.1186/s12913-018-2977-1, 2-s2.0-85043778303. PMC5856378 29544478

[7] Manojlovich M. , Environmental and Personal Predictors of Professional Nursing Practice Behaviors in Hospital Settings, 2003. 10.1097/NNA.0b013e3181f37e7d 20859101

[8] Woo B. F. Y. , Lee J. X. Y. , and Tam W. W. S. , The Impact of the Advanced Practice Nursing Role on Quality of Care, Clinical Outcomes, Patient Satisfaction, and Cost in the Emergency and Critical Care Settings: a Systematic Review, Human Resources for Health. (2017) 11, no. 1, 10.1186/s12960-017-0237-9, 2-s2.0-85029215203. PMC5594520 28893270

[9] Mick D. J. and Ackerman M. H. , Advanced Practice Nursing Role Delineation in Acute and Critical Care: Application of the Strong Model of Advanced Practice, Heart & Lung. (2000) 29, no. 3, 210–221, 10.1067/mhl.2000.106936, 2-s2.0-0034046231. 10819802

[10] Ackerman M. H. , Norsen L. , Martin B. , Wiedrich J. , and Kitzman H. J. , Development of a Model of Advanced Practice, American Journal of Critical Care. (1996) 5, no. 1, 68–73, 10.4037/ajcc1996.5.1.68. 8680496

[11] Chang A. M. , Gardner G. E. , Duffield C. , and Ramis M. A. , A Delphi Study to Validate an Advanced Practice Nursing Tool, Journal of Advanced Nursing. (2010) 66, no. 10, 2320–2330, 10.1111/j.1365-2648.2010.05367.x, 2-s2.0-77956292209. 20626481

[12] Gardner G. , Chang A. , and Duffield C. , Making Nursing Work: Breaking Through the Role Confusion of Advanced Practice Nursing, Journal of Advanced Nursing. (2007) 57, no. 4, 382–391, 10.1111/j.1365-2648.2007.04114.x, 2-s2.0-33846782809. 17291202

[13] Gardner G. , Duffield C. , Doubrovsky A. , and Adams M. , Identifying Advanced Practice: a National Survey of a Nursing Workforce, International Journal of Nursing Studies. (2016) 55, 60–70, 10.1016/j.ijnurstu.2015.12.001, 2-s2.0-84958183801. 26754956

[14] Carryer J. , Wilkinson J. , Towers A. , and Gardner G. , Delineating Advanced Practice Nursing in New Zealand: a National Survey, International Nursing Review. (2018) 65, no. 1, 24–32, 10.1111/inr.12427, 2-s2.0-85042119658. 29266216

[15] Jokiniemi K. , Bryant-Lukosius D. , Roussel J. et al., Differentiating Specialized and Advanced Nursing Roles: the Pathway to Role Optimization, Cjnl. (2023) 36, no. 1, 57–74, 10.12927/cjnl.2023.27123. 37552518

[16] Jokiniemi K. , Heikkilä A. , Meriläinen M. et al., Advanced Practice Role Delineation Within Finland: a Comparative Descriptive Study, Journal of Advanced Nursing. (2022) 78, no. 6, 1665–1675, 10.1111/jan.15074. 34655100

[17] Woo B. F. Y. , Zhou W. , Lim T. W. , and Tam W. W. S. , Practice Patterns and Role Perception of Advanced Practice Nurses: a Nationwide Cross‐Sectional Study, Journal of Nursing Management. (2019) 27, no. 5, 992–1004, 10.1111/jonm.12759, 2-s2.0-85064707618. 30776163

[18] Jokiniemi K. , Chair S. Y. , Wong F. K. Y. , and Bryant‐Lukosius D. , Advanced Practice Role Delineation Within Hong Kong: a Cross‐Sectional Study, Nursing and Health Sciences. (2022) 24, no. 3, 679–689, 10.1111/nhs.12964. 35699666 PMC9545430

[19] Nahari A. , Alhamed A. , Moafa H. , Aboshaiqah A. , and Almotairy M. , Role Delineation of Advanced Practice Nursing: a Cross‐Sectional Study, Journal of Advanced Nursing. (2024) 80, no. 1, 366–376, 10.1111/jan.15797. 37449552

[20] Sevilla Guerra S. , Miranda Salmerón J. , and Zabalegui A. , Profile of Advanced Nursing Practice in Spain: a cross-sectional Study, Nursing and Health Sciences. (2018) 20, no. 1, 99–106, 10.1111/nhs.12391, 2-s2.0-85042929976. 29235222

[21] Duffield C. , Gardner G. , Doubrovsky A. , and Adams M. , Does Education Level Influence the Practice Profile of Advanced Practice Nursing?, Collegian. (2021) 28, no. 3, 255–260, 10.1016/j.colegn.2020.08.006.

[22] Zumstein-Shaha M. and Grace P. J. , Competency Frameworks, Nursing Perspectives, and Interdisciplinary Collaborations for Good Patient Care: Delineating Boundaries, Nursing Philosophy. (2023) 24, no. 1. 10.1111/nup.12402 PMC10078421 35761762

[23] Williams G. A. and Kibowski F. , Jason L. A. and Glenwick D. S. , Latent Class Analysis and Latent Profile Analysis, 2025, Oxford University Press, New York.

[24] Wei W. , Hou Y. , and Yang Y. , A Study on International Experience Reference for Actively Responding to Population Aging, China Price. (2024) 2, 95–99.

[25] Lin W. , Zhang P. , Li S. et al., Category Characteristics and Influencing Factors of Research Competence Among Chinese Specialty Nurses: a Latent Profile Analysis, Journal of Advanced Nursing. (2025) . 10.1111/jan.16735 39791937

[26] Ministry of Health of China , Outline of the Development Plan for Nursing Care in China (2005-2010), Chinese Journal of Nursing. (2005) 40, no. 10, 721–723.

[27] Ding Y. , Wu X. , and Tian J. , A National Survey on the Training and Management of Specialist Nurses in Tertiary Hospitals Across 31 Provinces of China, Chinese Journal of Nursing. (2021) 56, no. 9, 1357–1362.

[28] Brown S. J. , A Framework for Advanced Practice Nursing, Journal of Professional Nursing. (1998) 14, no. 3, 157–164, 10.1016/s8755-7223(98)80091-4, 2-s2.0-0032059529. 9610024

[29] Taylor F. , Drennan V. M. , Halter M. , Allan H. T. , and Collins L. , Uptake of Advanced Clinical Practice Roles in the Health Service in England: Perspectives at the Micro Level, SSM-Qualitative Research in Health. (2022) 2, 10.1016/j.ssmqr.2022.100141. 34374583

[30] Higgins A. , Murphy R. , Downes C. , Varley J. , Begley C. , and Elliott N. , Factors Influencing the Implementation of Epilepsy Specialist Nurse Role: Using the Consolidation Framework for Implementation Research, Journal of Clinical Nursing. (2020) 29, no. 7–8, 1352–1364, 10.1111/jocn.15197. 31972049

[31] Rivera D. , Prades J. , Sevilla Guerra S. , and Borras J. M. , Contextual Factors Influencing the Implementation of Advanced Practice Nursing in Catalonia, Spain, International Nursing Review. (2024) 71, no. 2, 309–317, 10.1111/inr.12866. 37535808

[32] Tashakkori A. and Teddlie C. , Handbook of Mixed Methods in Social & Behavioral Research, 2003, SAGE.

[33] Gardner G. , Duffield C. , and Gardner A. , The Australian Advanced Practice Nursing Self-Appraisal Tool (The ADVANCE Tool), Figshare. (2017) .

[34] Zhao W. , Zhu Z. , and Shen Z. , The Chinese Version of the Advanced Practice Nursing Self-Appraisal Tool and Its Reliability and Validity Test, Chinese Nursing Research. (2024) 38, no. 24, 4378–4386.

[35] Braun V. and Clarke V. , Using Thematic Analysis in Psychology, Qualitative Research in Psychology. (2006) 3, no. 2, 77–101, 10.1191/1478088706qp063oa, 2-s2.0-33750505977.

[36] Chun C. K. , Wong F. K. , Wang S. L. , and Chen W. , Examining Advanced Nursing Practice in Hong Kong and Guangzhou, International Journal of Nursing Science. (2021) 8, no. 2, 190–198, 10.1016/j.ijnss.2021.03.001. PMC8105546 33997133

[37] Fosah R. and Llahana S. , Barriers and Enablers to Leadership in Advanced Practice Nursing: a Systematic Review, International Nursing Review. (2025) 72, no. 2, 10.1111/inr.70034. PMC12096811 40401735

[38] Heinen M. , van Oostveen C. , Peters J. , Vermeulen H. , and Huis A. , An Integrative Review of Leadership Competencies and Attributes in Advanced Nursing Practice, Journal of Advanced Nursing. (2019) 75, no. 11, 2378–2392, 10.1111/jan.14092, 2-s2.0-85074130937. 31162695 PMC6899698

[39] Doran D. , Duffield C. , Rizk P. , Nahm S. , and Chu C. H. , A Descriptive Study of Employment Patterns and Work Environment Outcomes of Specialist Nurses in Canada, Clinical Nurse Specialist. (2014) 28, no. 2, 105–114, 10.1097/nur.0000000000000031, 2-s2.0-84894189649. 24504037

[40] Soh B. F. J. R. , Ang W. H. D. , De Roza J. G. , Quek I. P. , Lim P. S. , and Lau Y. , They Are Partners in Care: a Qualitative Exploration of Physicians’ Perceptions of Primary Care Advanced Practice Nurses, Jounal of Nursing Scholarship. (2021) 53, no. 5, 542–551, 10.1111/jnu.12665. 33870641

[41] Petersen P. A. and Way S. M. , The Role of Physician Oversight on Advanced Practice Nurses’ Professional Autonomy and Empowerment, Journal of American Association Nurse Practice. (2017) 29, no. 5, 272–281, 10.1002/2327-6924.12444, 2-s2.0-85013481687. 28220626

[42] Ding Y. , Wu X. , and Wang X. , A Survey on Specialist Nurse Training by Nursing Associations at Municipal Level and Above in China, Chinese Journal of Nursing. (2020) 55, no. 5, 747–750.

[43] Gardner G. , Chang A. M. , Duffield C. , and Doubrovsky A. , Delineating the Practice Profile of Advanced Practice Nursing: a cross-sectional Survey Using the Modified Strong Model of Advanced Practice, Journal of Advanced Nursing. (2013) 69, no. 9, 1931–1942, 10.1111/jan.12054, 2-s2.0-84882281174. 23186155

[44] Park J. , Athey E. , Pericak A. , Pulcini J. , and Greene J. , To what Extent Are State Scope of Practice Laws Related to Nurse Practitioners’ Day-to-Day Practice Autonomy?, Medical Care Research and Review. (2018) 75, no. 1, 66–87, 10.1177/1077558716677826, 2-s2.0-85040659340. 29148318

[45] Kleinpell R. , Myers C. R. , Likes W. , and Schorn M. N. , Breaking down Institutional Barriers to Advanced Practice Registered Nurse Practice, Nursing Administration Quarterly. (2022) 46, no. 2, 137–143, 10.1097/naq.0000000000000518. 35239584

[46] Boehning A. P. and Punsalan L. D. , Advanced Practice Registered Nurse Roles, StatPearls [Internet], 2025, StatPearls Publishing, Treasure Island (FL). 36944002

[47] Lawrence K. and Motta G. , Reimbursement Opportunities for WOC Nursing Services: Medicare Part B “Incident To” Services Policy: a Fact Sheet, Journal of Wound Ostomy Continence Nursing. (2019) 46, no. 4, 351–353, 10.1097/won.0000000000000550, 2-s2.0-85069268785. 31274871

